# Epigenetic Targets for Oligonucleotide Therapies of Pulmonary Arterial Hypertension

**DOI:** 10.3390/ijms21239222

**Published:** 2020-12-03

**Authors:** William Gerthoffer

**Affiliations:** Department of Pharmacology, Reno School of Medicine, University of Nevada, Reno, NV 89557, USA; wgerthoffer@med.unr.edu; Tel.: +1-775-229-3028

**Keywords:** DNA methylation, histone code, microRNA, nanoparticles, noncoding RNA, pulmonary arterial hypertension

## Abstract

Arterial wall remodeling underlies increased pulmonary vascular resistance and right heart failure in pulmonary arterial hypertension (PAH). None of the established vasodilator drug therapies for PAH prevents or reverse established arterial wall thickening, stiffening, and hypercontractility. Therefore, new approaches are needed to achieve long-acting prevention and reversal of occlusive pulmonary vascular remodeling. Several promising new drug classes are emerging from a better understanding of pulmonary vascular gene expression programs. In this review, potential epigenetic targets for small molecules and oligonucleotides will be described. Most are in preclinical studies aimed at modifying the growth of vascular wall cells in vitro or normalizing vascular remodeling in PAH animal models. Initial success with lung-directed delivery of oligonucleotides targeting microRNAs suggests other epigenetic mechanisms might also be suitable drug targets. Those targets include DNA methylation, proteins of the chromatin remodeling machinery, and long noncoding RNAs, all of which act as epigenetic regulators of vascular wall structure and function. The progress in testing small molecules and oligonucleotide-based drugs in PAH models is summarized.

## 1. Introduction

**Figure 1:**
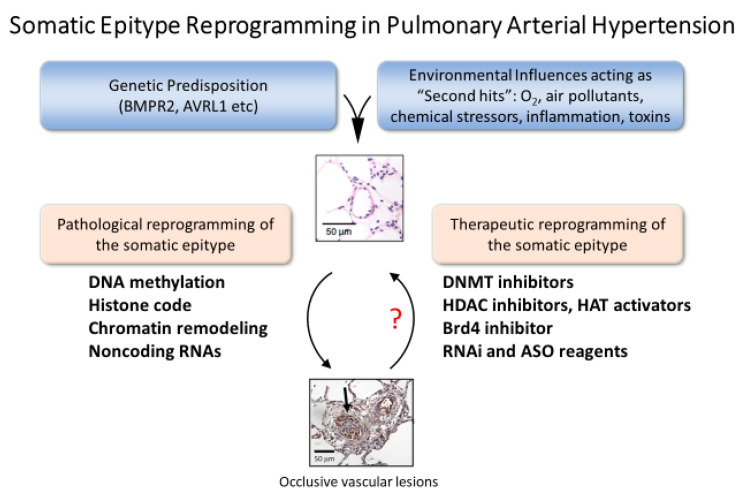
Somatic epitype reprogramming as a therapeutic strategy in PAH. Vascular wall cells involved vascular remodeling are all potential targets for novel epigenetic therapies. Both inflammatory and structural cells of the vascular wall contribute to remodeling in pulmonary hypertension. The therapeutic goal is to modify gene expression programs to reverse and prevent occlusive lesions. The contributions of epigenetic processes to the function of each cell type in the lung is a topic of intense interest and rapid progress in lung cell biology.

The set of proteins expressed and the abundance of proteins in cells of the vascular wall is a complex function of transcription, translation, and regulation of protein lifespan. In addition to the heritable protein-coding sequences in DNA, there are epigenetic processes in somatic cells that modify gene expression within a generation to define the somatic epitype (Figure 1) [1]. In this way, the normal gene expression programs and pathological gene expression programs are modulated during development and during adaptation to variations of cellular and organ homeostasis. Diseases that change the extracellular milieu will trigger changes in phenotype in part by activating epigenetic processes. For example, DNA methylation typically inhibits transcription, thus silencing genes not expressed in a given tissue under a given set of conditions. DNA methylation occurs at CpG islands, often in promoters of protein-coding genes. Posttranslational modifications of histones act in tandem with DNA methylation to either repress or permit transcription by opening chromatin to allow access of the transcriptional machinery. Transcription produces several species of protein-coding (mRNA, some circRNAs) and noncoding RNAs (miRNA, lncRNA, circRNAs) that can modulate transcription, mRNA processing, and mRNA stability. The net effect of these epigenetic processes is to regulate protein abundance, which is the defining molecular component of phenotype. Epigenetic processes are highly conserved, occurring in all the cells of the pulmonary circulation. This suggests vascular arteriopathy of pulmonary artery hypertension might respond to drugs that alter one or more epigenetic process. However, there is significant heterogeneity of DNA methylation patterns, histone marks, and noncoding RNA expression patterns among cell types.

Epigenetic therapies could be developed that are effective with tolerable off-target effects. To establish appropriate drug targets, the timing and the necessity for particular epigenetic adaptations in developing pulmonary hypertension need to be defined. Many new drug targets have been described in preclinical studies which are summarized in this review and in several recent reviews of the same topic [2,3,4,5,6]. The premise is that drugs antagonizing both causative and adaptive epigenetic events will prevent disease progression and allow vascular wall repair, thus favoring a return to normal vascular structure. Drug therapy begins well after PAH has developed, often after several years of onset of symptoms. This means new drugs must reverse vascular remodeling to become clinically useful. Several aspects of pulmonary vascular remodeling are of particular interest in this regard—vascular wall thickening, formation of occlusive lesions, and vascular pruning. In this review, some of the landmark studies of epigenetic modifiers in PAH are described along with some more recent work on chromatin remodeling, including BET proteins and noncoding RNAs. Novel oligonucleotide tools and therapeutics are emerging from this very exciting new work. The reader should consult earlier reviews for a general appreciation of advances in epigenetic therapy in other organ systems [7,8,9,10].

Targeting epigenetic mechanisms is appealing because enzymatically catalyzed reactions are often reversible, and the enzymes are targets for small molecules and oligonucleotide drugs. This is in sharp contrast to mutations in genomic and mitochondrial DNA, which are not routinely correctable in a clinical setting, although remarkable advances in gene editing technology have created new opportunities that are advancing rapidly [11]. This review will describe recent progress in preclinical studies of small molecule and oligonucleotide modifiers with the goal of focusing on new therapeutic approaches that may add to current treatments of PAH.

## 2. Pulmonary Vascular Remodeling in PAH

Pulmonary arterial hypertension (WHO Group I) is a rare, progressive pulmonary vascular disease characterized by elevated pulmonary artery pressure (>25 mm Hg), dyspnea, exercise intolerance, and ultimately right heart failure. In the USA, it is more common in women than men (3–5:1) and in Caucasians (85%) compared to African-Americans (12%) and Hispanics (<3%) [12]. The pathological vascular remodeling in PAH is the result of increased proliferation and decreased apoptosis of vascular wall cells, including smooth muscle, endothelial cells, fibroblasts, and immune cells. Vascular wall hypertrophy is associated with increased inward migration of progenitor cells and immune cells, as well as altered autophagy and cell differentiation. These processes are the subject of intense research to understand basic pulmonary vascular wall cell biology and to identify novel targets for drug development [13,14,15]. In addition to identifying novel inhibitors of cell signaling, noncoding RNAs (miRNA and long noncoding RNAs) have undergone intense scrutiny as molecular targets for anti-remodeling therapy [16]. In addition, early evidence of changes in DNA methylation [17] and histone marks [18] in pulmonary hypertension stimulated an active search for targets for new therapies [19,20].

**Figure 2:**
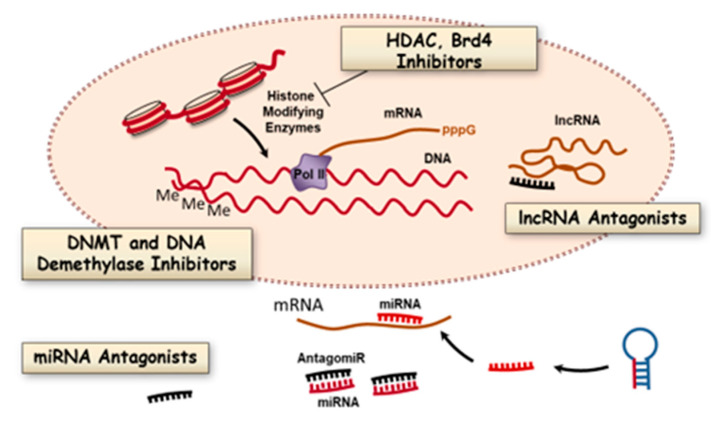
Epigenetic processes that are potential targets for modifying pulmonary vascular remodeling in PAH. There are multiple biochemical processes that promote arteriopathy in PAH by altering gene expression programs in vascular wall cells. These processes are valid therapeutic targets for small molecule inhibitors of DNA methylation and histone modifications and other elements of chromatin remodeling machinery. In addition, oligonucleotide antagonists of short and long noncoding RNAs (miRNAs, lncRNAs) are also potential new classes of antiremodeling drugs.

There are several issues to address when developing therapies to reprogram the somatic epitype in order to prevent or reverse vascular remodeling. Which processes in which cells should be targeted (Figure 2)? What are the epigenetic drivers of pulmonary remodeling vs. the adaptive responses to disease (Figure 2)? Perhaps most importantly, are there unique targets that produce disease-specific effects such as regulation of bone morphogenetic protein receptor 2 (*BMPR2*) expression and function in subjects with familial PAH? In pulmonary vascular remodeling and repair, the druggable targets include enzymes that catalyze DNA methylation and demethylation, enzymes of DNA repair pathways, enzymes that catalyze histone posttranslational modifications, and noncoding RNAs. Most studies described below are early-stage preclinical trials arranged by biochemical and molecular classes.

## 3. Epigenetic Targets for Novel Therapy of PAH 

### 3.1. DNA Methylation and Inhibitors

One of the major determinants of whether a gene is transcribed is methylation of DNA, typically but not exclusively in CpG islands in promoter regions (Figure 2). DNA methylation is catalyzed by DNA methyltransferase (DNMT) and removed by demethylases. These classes of enzymes have been studied extensively in cancer chemotherapy [21], but less thoroughly in PAH. Although the data on DNA methylation patterns in lung diseases other than lung cancer are less extensive, some interesting patterns have emerged that are relevant to PAH. A landmark study by Archer and coworkers reported DNA methylation patterns in the superoxide dismutase gene in vascular smooth muscle cells in the Fawn hooded rat model of PAH [17]. Aside from this study, there is no direct evidence for changes in DNA methylation or hydroxymethylation and gene expression in endothelial cells, immune cells, or fibroblasts in PAH. However other studies of related conditions might be relevant to future work. For example, increased methylation of the granulysin gene (*GLYN*) occurs in peripheral blood monocytes of human subjects with pulmonary veno-occlusive disease, but not in patients with PAH [22]. The authors concluded that *GLYN* genes in several T-cell populations (cytotoxic, natural killer and natural killer-like T cells) were not altered in PAH. This suggests some disease-restricted effects on immune cell gene silencing that offer selective targets for drugs altering DNA methylation. Further work in PAH samples is required to find analogous PAH-restricted methylation patterns.

Methylation of the *BMPR2* promoter in scleroderma patients is another relevant observation [23]. Patients with scleroderma may be predisposed to PAH due to *BMPR2* promoter methylation and reduced BMPR2 expression. Later studies of *BMPR2* silencing by DNA methylation in PAH patients are mixed. One study of peripheral blood cell DNA reported no methylation of the *BMPR2* promoter [24], but a more recent study did find increased methylation and reduced expression of BMPR2 protein in heritable PAH [25]. Several mutations of the Tet-methylcytosine-dioxygenase-2 (*TET2)* gene coding for a DNA demethylase have been reported in humans with PAH. Furthermore, *TET2* knockout mice develop a mild form of PAH [26]. These later studies support the significance of dynamic changes in DNA methylation and suggest additional genes might be worth investigating. For example, there is no information on DNA 5mC and 5hmC methylation status of proinflammatory genes in humans with PAH. There is significant infiltration of immune cells into occlusive vascular lesions in humans and in animal models of PAH, but no knowledge of inflammatory gene silencing by DNA modifications. This seems likely given that a genome-wide association study (GWAS) of systemic hypertension found several loci where DNA methylation patterns were associated with hypertension [27]. Similar genome-wide serial DNA methylation studies could be conducted in models of severe PAH models to establish patterns of altered 5mC and 5hmC patterns. Such a study in humans would be challenging due to the low prevalence of PAH and the inability to conduct a longitudinal study of diseased arterial tissue. Despite these limitations, the loci identified in human studies of systemic hypertension might serve as a guide to studies in animal models of severe PAH.

The timing of a therapeutic intervention that reduces DNA methylation will be important to establish. If changes in DNA methylation occur prior to diagnosis (drivers) the damage may be difficult to reverse versus ongoing DNA methylation during the progression of the disease (adaptive responses). It is not clear whether DNA methylation can be selectively modulated with drugs, but there is some reason for optimism. De novo DNA methylation is dynamic and reversible by the action of demethylases. Blocking DNMT activity might be effective in allowing vascular repair, as shown by Archer and coworkers using 5-azacytidine in a rat model of PAH [17]. This study has an important limitation in that 5-azacytidine has pharmacological effects in addition to DNMT inhibition [28]. More selective agents must be developed, preferably with some lung-restricted distribution to minimize off-target effects. Targeting DNA methylation machinery with oligonucleotide-based drugs is an approach tested in cell systems and with knockout mouse models. Several oligonucleotides targeting elements of DNA methylation have been tested as treatments of neurological diseases. Targets include DNMTs 1 and 3 a/b and Tett1 [29,30,31]. However, similar studies have not been attempted in animal models of pulmonary hypertension. Delivery of oligonucleotides to lung tissues is well established as described below, which suggests that altering the DNA methylation/demethylation machinery might be achievable.

### 3.2. Histone Modifications and Inhibitors 

#### 3.2.1. Histone Deacetylases 

Post-translational modifications of histones control chromatin structure by charge effects and by recruiting additional chromatin remodeling enzymes [32]. In general, lysine acetylation of the histone tails permits transcription. Deacetylation is restrictive, but the effects vary with the particular gene being regulated. Histone acetylation is catalyzed by histone acetyltransferases (HATs) and histone deacetylation by a large family of protein deacetylases (HDACs and sirtuins). Histone marks are modified during normal development and often in disease. The roles in development and diseases have been explored in detail using numerous small molecule inhibitors of protein acetylases and methylases that catalyze histone modification [33]. Several of these have been tested as drugs to modify vascular remodeling, as described in more detail below. Methylation of histones can also be either permissive or restrictive depending upon the methylated residue. Two of the best-studied examples are H3K4 di/tri-methylation, which is permissive, and H3K9 di/tri-methylation, which is restrictive. Histone methylation is catalyzed by histone lysine or arginine methyltransferases. Histone demethylases catalyze the reverse reactions. Some serine residues on histones are phosphorylated, but this topic is less well developed compared to histone methylation and acetylation. There is also emerging evidence for modification of glutamine 5 in histone 3 (H3Q5) with serotonin and dopamine in neuronal tissues, but there is no evidence yet of these interesting modifications in vascular wall cells [34,35]. Disease-restricted alterations in the histone code are the results of changes in activity, chromatin binding, or expression of histone-modifying enzymes. Imbalances in the activity of these enzymes and resulting changes in open versus closed chromatin states are associated with numerous diseases, including pulmonary arterial hypertension (see Figure 2 above).

A growing set of pharmacological studies implicate histone acetylation in PAH by virtue of the effects of HDAC inhibitors on vascular remodeling and cardiac function. Detailed progress at the molecular level is somewhat limited by the modest understanding of exactly which genes to focus on. Some of the earliest evidence supporting HDACs in pulmonary hypertension is from a study of the bovine model of hypoxic pulmonary hypertension [36]. Apicidin, a class I HDAC inhibitor, reduced proinflammatory gene expression in pulmonary adventitial fibroblasts from chronically hypoxic calves. SAHA, a broad spectrum HDAC inhibitor, reduced fibroblast induced migration of monocytes, suggesting that HDACs support vascular inflammation and that HDAC inhibitors can be anti-inflammatory drugs in treating PAH. This begs the question of whether HDAC expression and activity is altered in humans with PAH. In humans with heritable PAH, increased expression of HDACs 1, 4, and 5 was observed, as was a predicted increase in H3 and H4 acetylation [37]. In the same study, valproic acid and SAHA prevented vascular remodeling in a hypoxic rat model.

Inhibiting HDACs in PAH might be beneficial in part because it alters ROS production. In macrophages and THP-1 cells, HDAC inhibitors reduced NOX 1, 2, and 4 expression of macrophages and THP-1 cells by reducing Pol II and p300 HAT loading on *NOX* gene promoters [38]. H3K4me3 and H3K9ac histone marks were also reduced by HDAC inhibition in lung fibroblasts. In pulmonary endothelial cells, there is evidence for HDAC4 and HDAC5 regulating Mef2 activity, which participates in a signaling cascade that influences cell migration and proliferation [20]. The class IIa HDAC inhibitor MC1568 reversed signs of PAH in several rat models, suggesting HDACs 4 and 5 contributed to pulmonary artery endothelial dysfunction. This result is part of an increasing set of studies of HDAC inhibitors in rat models that suggest HDAC inhibitors have beneficial prevention and reversal effects [20,37]. Some of these effects are mediated by nuclear HDACs, but there is also evidence for nonnuclear HDAC6 acting on substrates other than histones to affect pulmonary vascular remodeling [39]. Studies of multiple HDAC inhibitors in multiple PAH models suggest that regulation of proinflammatory genes, pro-growth genes, and promigratory signaling genes are regulated by histone modifications that may well initiate and promote arteriopathy and control PAH severity. Further preclinical studies and mechanistic studies are needed to refine the use of HDAC inhibitors with a goal of identifying the HDAC isoforms to target for the most effective therapy of PAH. In contrast to the significant literature on small molecule HDAC inhibitors in animal models of PAH, there are few studies of oligonucleotides targeting HDACs in vascular tissues.

#### 3.2.2. Histone Acetyltransferases

There are only a few studies of histone acetyltransferases in PAH. It is known that histone acetyltransferase activity is higher in lung tissue of PAH patients [40], but it is less clear that increased histone acetylation promotes inflammation, proliferation, cell migration, or cell survival in the arterial wall. It appears some of the benefits of prostacyclin therapy may be due to modifying histone marks and reducing inflammation. Iloprost decreased secretion of several pro-inflammatory proteins (IL-8, CCL2, RANTES and TNFα) in activated human monocytes [41]. Reduced cytokine production correlated with decreased STAT1 phosphorylation and with decreased localization of the p300 HAT at the STAT1 promoter. This is an interesting observation, but more information is needed to define the proinflammatory genes sensitive to HAT inhibitors and to establish the therapeutic significance of HAT inhibitors for reversing arteriopathy of PAH.

#### 3.2.3. Histone Methylation

Methylation of histones has a cooperative effect with DNA methylation to favor heterochromatin formation and gene repression [42]. During embryonic development, SET histone methyltransferases catalyze H3K9 trimethylation, which enhances DNA methylation by DNMT3a and DNMT3b enzymes [42]. When somatic cells undergo reprogramming during neoplastic transformation, changes in DNA methylation of pluripotency genes are guided in part by histone demethylation. This suggests changes in histone H3K9 methylation might influence vascular remodeling and perivascular inflammation in PAH. To address this question, a recent study investigated the influence of the Nuclear receptor binding SET domain 2 (NSD2) histone methyltransferase. NSD2 is upregulated in some cancers and cancer models where it promotes somatic reprogramming and transformation [43]. In the monocrotaline rat model, knockdown of NSD2 with shRNA reduced H3K36 dimethylation and antagonized pulmonary arterial remodeling, normalized pulmonary artery pressure, and normalized right ventricular dysfunction [44]. Metabolomic analysis suggested NSD2 regulates genes controlling autophagy, carbohydrate metabolism, transporters, and protein synthesis. This is a novel role for histone methylation in PAH that provides an interesting new target for drug therapy. There are a number of small-molecule NSD2 inhibitors in development as anticancer chemotherapy, but no oligonucleotide-based drugs have been reported in human studies. Both approaches might be useful for knocking down NSD2 in vivo in PAH models, although the transfer of cancer chemotherapy approaches to PAH is not always a useful therapeutic strategy. Further preclinical development of NSD2 inhibitors in PAH models is required to address safety and efficacy in nonneoplastic diseases.

### 3.3. Bromodomain Proteins

Bromodomain-containing proteins bind acetylated lysine residues on histones and function as transcriptional coactivators in the histone code “reader” machinery [45,46]. Vascular remodeling in PAH depends on changes in the transcription of multiple pathways in multiple cell types. One the most important transcriptional regulators is NFĸB, particularly for genes expressed during vascular inflammation. NFĸB-regulated genes are also dynamically controlled in normal human pulmonary vascular endothelial cells, as shown by cell cycle arrest induced by the BET inhibitor JQ1 [40]. JQ1 increased expression of proteins p19INK4D and p21CIP1 and reduced expression of cyclin-dependent protein kinases. JQ1 treatment also increased HAT activity, presumably due to reducing the association of BET proteins with HATs in histone reader/writer complexes. BET inhibition also inhibits the proliferation of pulmonary artery vascular smooth muscle cells [47]. In both endothelium and smooth muscle cells, BRD4 was the proposed target of JQ1. It appears that BET inhibitors used in a rescue strategy have significant beneficial anti-inflammatory effects in animal models of PAH [48], which is the rationale for a phase 2 clinical trial of apabetalone, an orally available BRD4 inhibitor (ClinicalTrials.gov Identifier: NCT03655704). Recent reports of cardiac toxicity temper enthusiasm for broad-spectrum BET inhibitors [49]. Isoform-selective drugs and lung-restricted delivery might be necessary to minimize adverse effects on non-targeted organs [48].

In contrast to the significant literature on small molecule inhibitors of histone remodeling in PAH animal models, there is a lack of studies of oligonucleotides targeting HDACs, HATs, or BET proteins in vascular tissues. Although there are numerous antisense oligonucleotides available to modify chromatin remodeling machinery in vitro, there are few or no preclinical drug development studies of PAH models. Drug delivery and tissue specificity are two important problems slowing the development of new oligonucleotide drugs. These two issues have been addressed to some extent by recent studies of oligonucleotides targeting microRNAs in animal models of PAH.

### 3.4. MicroRNAs

**Figure 3:**
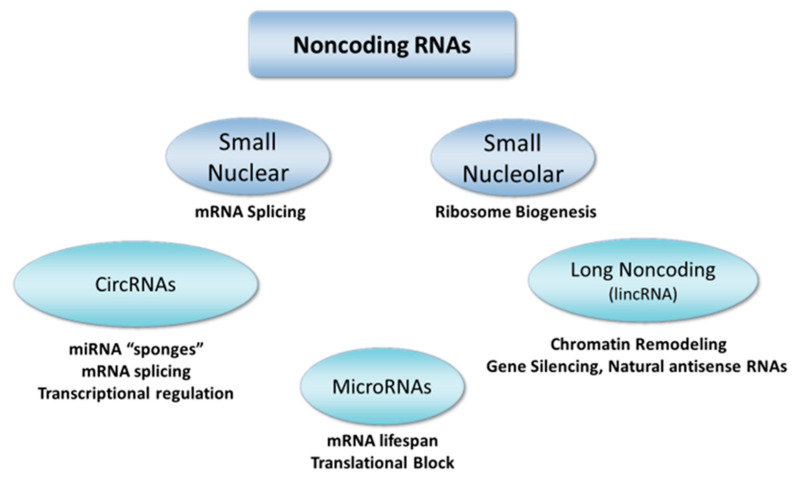
Classes of noncoding RNAs. Cell growth, organ development, and pathological remodeling all depend on dynamic changes in the expression of noncoding RNAs. MicroRNAs are the best-established class of noncoding RNAs with important roles in regulating the translation of proteins that remodel the pulmonary vasculature. Long noncoding RNAs are also modified during the development of PAH, but their functions are less clear. Circular RNAs are emerging as collaborators with the other classes of RNAs to regulate mRNA production, transcription, and gene silence via interactions with miRNAs. miRNAs, long noncoding RNAs, and circular RNAs are all potentially targetable molecules using lung-directed delivery of oligonucleotide antagonists and mimics.

Several classes of noncoding RNAs regulate gene expression and protein abundance to fine-tune cell phenotypes in conditions of health and disease (Figure 3). MicroRNAs, long noncoding RNAs, and circular RNAs are all modulators of steps in the flow of genetic sequence information from genomic DNA to mRNA and to proteins. Transcription of mRNA depends on the synthesis of adequate numbers of ribosomes. Ribosome biogenesis requires a variety of small nucleolar RNAs that are structural elements of ribosomes, as well as small nuclear RNAs that participate in mRNA splicing and processing of mature mRNA. Once processed and associated with ribosomes, mature mRNAs are translated to peptides by complex biochemical machinery subject to extensive regulation. MicroRNAs fine-tune the output of gene expression programs by modulating translation, thus varying protein abundance and cell phenotype.

Numerous correlative studies of miRNA expression in vascular tissue from humans have defined altered miRNA expression patterns in PAH. The miR-17~92 cluster, MiR-21, miR-145, and miR-204 were the first miRNAs associated with PAH [50,51,52,53,54] (Table 1). Similar lists of miRNAs altered in animal models have also been assembled and are described in prior reviews [4,55,56]. Early correlative studies stimulated preclinical translational studies designed to normalize miRNA expression and to halt or reverse the progression of PAH. There is now substantial proof of the efficacy of RNAi-based therapies targeting miRNAs in PAH. Preclinical prevention trials in animal models of PAH have described the roles of miR-204 [52], miR-17 [57], miR-21 [58], miR-20a [59], miR-145 [53,60], miR-223 [61], miR-424, and miR-503 [62] in PAH pathology.

**Figure 4:**
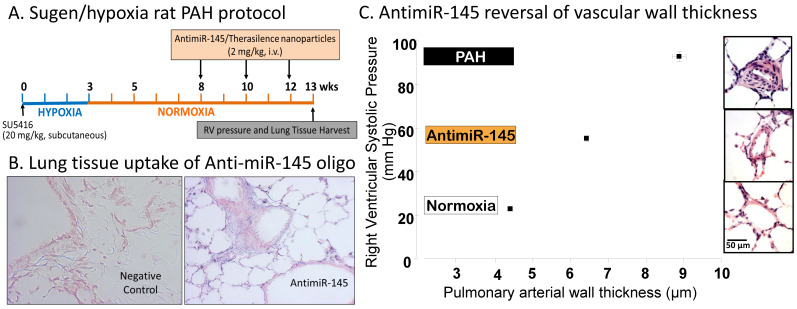
A rat model of severe PAH employed treatment with Sugen5416 followed by a period of hypoxia. Right ventricular systolic pressure (RVSP) increased to >90 mmHg at 13 weeks (**A**). Lung tissue uptake of the antimiR-145 antisense oligonucleotide was assessed by in situ hybridization (**B**), blue staining). (**C**) shows a positive correlation of RVSP with vessel wall thickness in small pulmonary arteries (50–200 µm dia.). AntimiR-145 treatment partially reversed both wall thickening and RVSP. Data are replotted from McLendon et al. [60].

One limitation of initial studies of miRNA mimics and antagonists is that none used intravenous therapy to achieve lung-directed delivery. They also used prevention protocols rather than a rescue protocol to reverse severe occlusive vascular remodeling. This limitation is relevant to drug therapy because humans are diagnosed with PAH after it is well established, not prior to the development of arteriopathy and cardiac dysfunction. A later reversal study [60] employing a pegylated cationic lipid nanoparticle (Therasilence) delivered intravenously showed that a miR-145 antagonist could ameliorate many pathological features of established PAH in the Sugen/hypoxia rat model of severe PAH (Figure 4A). The miR-145 antisense oligonucleotide was a locked nucleic acid/DNA mixmer that achieved high levels in lung tissues (Figure 4B). AntimiR-145 treatment partially reversed vascular wall thickening (Figure 4C). Right ventricular systolic pressure was reduced, and there was a modest reduction of perivascular inflammation. Similar anti-inflammatory effects were reported in another reversal study by Gubrij et al. [61] using a miR-223 antagonist in the monocrotaline rat model of PAH. The studies summarized in Table 1 include both prevention trials and reversal trials in animal models showing that therapeutic oligonucleotides targeting microRNAs can be delivered to the lung via the airway or from the vascular compartment to ameliorate vascular inflammation, vascular medial thickening, and occlusive vascular lesions in PAH.

One question that remains unanswered is which cell types should be targeted in treating PAH with oligonucleotide drugs? Many mechanistic studies of miRNAs in pulmonary artery remodeling have focused on endothelial cells and vascular smooth muscle cells. However, some miRNAs associated with PAH in humans (miR-145, miR-155, miR-21 and the miR-17~92 cluster) also regulate inflammation and immune cell biology. For example, IL-6 upregulates expression of the miR-17~92 cluster, which targets BMPR2, suggesting that inflammation exacerbates PAH by inhibiting BMPR2 signaling. MiR-145 is known to regulate cell fate during early embryogenesis and is a master regulator of smooth muscle contractile phenotype. It also regulates the immune response by promoting M1 to M2 macrophage polarization [76] and Th2 cell development in the airways and thoracic lymph nodes [77]. MiR-21 is pro-inflammatory in mouse models of severe asthma [78]. MiR-124 silences MCP1 expression in adventitial fibroblasts, which probably influences the extent of vascular inflammation [79]. Future translational studies of miRNA antagonists could be designed to target multiple protein networks known to mediate vascular wall remodeling and perivascular inflammation. MicroRNA antagonists and mimics with combined vasodilating, anti-inflammatory, and anti-remodeling effects would constitute a novel class of therapy to complement and improve the efficacy of current vasodilator therapy. However, early-phase clinical trials have not yet been conducted on human subjects. One limitation is the complexity of drug delivery to the lungs. Complexing oligonucleotide drugs with carriers such as lipid nanoparticles or carbohydrate polymers clearly works in preclinical animal models. However, the delivery system may have pharmacological effects unrelated to the oligonucleotide target, which adds significant barriers to regulatory approval for use in humans.

### 3.5. Long Noncoding RNAs 

**Figure 5:**
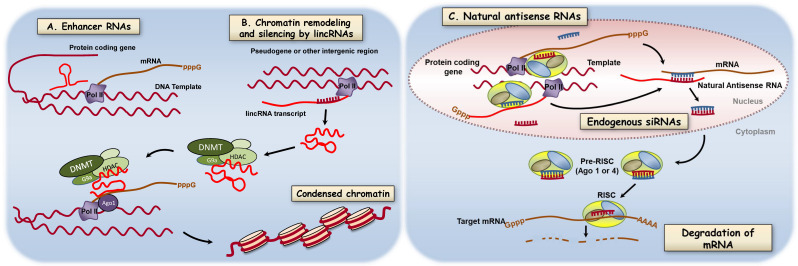
Regulation of gene expression by long noncoding RNAs. Long nonprotein coding RNAs are transcribed from a variety of genomic loci including enhancer sequences (eRNAs), intergenic, multiexomic sequences (lincRNAs), and noncoding strands of protein-coding genes (naturally occurring antisense transcripts. Shown here are two of the emerging mechanisms by which lncRNAs modify gene expression and protein abundance. (**A**) Binding of RNAs coded in enhancer regions to effect DNA looping. (**B**) lncRNA serving as an adapter molecule in chromatin remodeling via DNA methylation and histone modifications. A salient feature of lncRNAs is the potential to bind chromatin remodeling proteins, DNA, and in some cases other RNAs. (**C**) Some long noncoding RNAs are processed to endogenous siRNAs that regulate transcription or regulate translation by altering mRNA decay. Some long noncoding RNAs also modify mRNA splicing (not shown). Targeting new oligonucleotide drugs to lncRNA function will require identification of key lncRNAs in lung diseases and defining the contribution of these mechanisms to pathology. Reproduced from Comer et al. [55] with permission from Elsevier.

Long RNAs (>200nt) not translated to proteins (lncRNA) are transcribed from a variety of sites in the nuclear genome. There are lncRNAs from enhancer sequences of protein-coding genes (eRNAs) [80], RNAs from intergenic, multiexomic regions (lincRNAs) [81], and from noncoding strands of protein-coding genes (naturally occurring antisense transcripts, NATs) [82,83] (see Figure 5). The term long noncoding RNAs (lncRNA) will refer to all RNAs > 200 nt that do not code for proteins. The functions of most long noncoding RNAs are still being defined. Some are thought to serve as adapter molecules able to bind proteins, DNA, and other RNAs. This versatile set of binding reactions underlies some of the functions of lncRNAs which include: Regulation of transcription by controlling gene looping [80,84], regulation of mRNA splicing [85], regulation of mRNA lifespan [63,86], and organization of gene neighborhoods [87] (Figure 5). Recent work in various PAH models describe the influence of lncRNAs on microRNA function [73,88], on scaffolding functions [67], and on vascular cell proliferation and differentiation [63,64,65,74] (Table 1). Collectively, these studies strongly suggest lncRNAs are valuable targets for further translational drug development.

Although recent observational studies reveal the association between lncRNAs and PAH, it will be important to determine what strategies are used to modify lncRNA expression and function in the pulmonary circulation. Antisense oligonucleotides (“antagoNATs”) that inactivate lncRNAs in cells and in animal models of disease have been proposed [89] and used effectively in several of the recent studies of PAH cited above. The response to a lncRNA antagonist may be to increase protein expression if the lncRNA is a repressor of gene expression, or to reduce protein expression if the lncRNA is an enhancer. For example, inhibiting the function of MALAT1 reduces endothelial cell differentiation in vitro [90]. A MALAT1 antagonist active in vivo might reduce occlusive lesions in pulmonary arterial hypertension or inhibit airway epithelial cell proliferation. This notion is supported by the fact that polymorphisms in the *MALAT1* gene have been described in humans with essential hypertension [72]. MALAT1 reduced miR-539-3p and miR-485-3p levels and increased expression of BMP receptor type 2. The authors suggested low plasma levels of MALAT1 correlated with increased miRNA expression and reduced BMPR2 expression, conditions associated with PAH. Other lncRNAs might be targeted by oligonucleotides to inhibit lncRNAs that act as “sponges” for miRNAs. In this use case, the drugs would increase the expression of proteins repressed by the miRNA binding partners. The lncRNAs and proteins targeted might be proteins with anti-inflammatory effects [91], antiproliferative effects, or antimigratory effects. Another interesting strategy that remains untested is to disrupt lncRNA function with small molecules that bind to RNA secondary structure or disrupt the tertiary structure of ribonucleoprotein complexes [92].

## 4. Summary and Future Directions

The prospect of modifying epigenetic processes to alter the interaction of genes and the environment in pulmonary vascular cells is exciting and important. The premise is that cell phenotype and vessel architecture are determined by reversible biochemical processes acting on the genetic substrate. This is the mechanistic basis for several anticancer drugs that modify the epigenetic processes described in this review. In cancer chemotherapy, single epigenetic modifying drugs and combination therapies that include epigenetic modifiers are effective in some cases, but ideal drug regimens are still being defined. Successful epigenetic reprogramming in lung cancer chemotherapy is very encouraging and highly relevant to epigenetic therapy for PAH [93,94]. If normal control of cell proliferation, cell survival, and tissue boundaries is compromised, then vascular tissue growth might be reversible with drugs that reestablish normal epigenetic processes, normal cell phenotype, and normal tissue architecture. However, treating a nonneoplastic lung disease such as PAH with oligonucleotide drugs targeting epigenetic mechanisms is a relatively new enterprise. Repurposing anticancer drugs in PAH has produced mixed results but the idea that epigenetic reprogramming can be affected with drugs is worth pursuing. Most work is in vitro or in early preclinical stages. Recent progress in early-stage clinical trials is summarized in several reviews [95,96].

There are still significant gaps in knowledge that limit the development of epigenetic modifying oligonucleotide drugs. We need better descriptions of which miRNAs, lncRNAs, circRNAs, histone modifications, and DNA methylation sites are present at the single-cell level in PAH. We also need important longitudinal studies of epigenetic features in individual pulmonary vascular wall cells and in key genes controlling cell phenotype [2]. Serial genome-wide assays are needed to define which epigenetic events are causes and which are consequences of the disease. This would be analogous to GWAS studies of genomic DNA sequences, the key difference being that epigenetic modifications are dynamic and reversible. Therefore, longitudinal data similar to data used to validated prognostic biomarkers are needed to establish the best targets and the best timing of dosing. The function of the epigenetic marks and the pathways regulated then need to be validated in both human samples and in multiple clinically relevant animal models. Once high-value targets are identified, candidate drugs must be validated in preclinical animal trials and ultimately in initial clinical trials to establish efficacy, lung-directed delivery, and off-target effects. Potential adverse effects of oligonucleotide therapies that would appear in phase 2 clinical trials are difficult to predict. They will depend on the targets and on pharmacologic effects unrelated to the targets. For example, targeting conserved DNA and histone-modifying enzymes with broad-spectrum drugs that are widely distributed can disrupt organ function well beyond the lungs—e.g., in the bone marrow, heart, liver, kidneys or nervous system. Small oligonucleotides are also perceived by the immune system as damage-associated or pathogen-associated molecular patterns which can activate the innate immune response to deleterious effect [97,98]. Managing the off-target effects of oligonucleotide drugs is a challenge that needs to be overcome. However, if effective and safe oligonucleotides can be delivered preferentially to the lungs, they could become important adjuncts to vasodilator therapies that could act as true disease-modifying therapies.

## Figures and Tables

**Table 1:** Noncoding RNA targets in pulmonary arterial hypertension.

miRNAs	Long Noncoding RNAs
let-7f/miR-22/30 [51]	CASC2 [63]
miR-17/92 cluster [50,57]	H19 [64]
miR-21 [51]	Hoxaas3 [65]
miR-23b/130a/191/451/1246 3 [66]	HOXB1, CBL, GDF7, RND1 [67]
miR-124 [68]	LnRPT [69,70]
miR-143 [54]	MALAT1 [71], [72]
miR-145 [53]	Tug1 [73]
miR-204 [52]	UCA1 [74]
miR-206 [75]	
miR-322/451 [51]	
miR-424/503 [62]	

## Abbreviations

DNMT	DNA methyltransferase
HAT	histone acetyltransferase
HDAC	histone deacetylase
lncRNA	long noncoding RNA
MALAT1	metastasis associated lung adenocarcinoma transcript 1
miRNA	microRNA
NSD2	nuclear receptor binding SET domain 2
PAH	pulmonary arterial hypertension
RVSP	right ventricular systolic pressure
